# An OLD protein teaches us new tricks: prokaryotic antiviral defense

**DOI:** 10.1038/s41467-024-46925-1

**Published:** 2024-03-21

**Authors:** Eirene Marie Q. Ednacot, Benjamin R. Morehouse

**Affiliations:** 1 Department of Molecular Biology and Biochemistry, School of Biological Sciences, University of California Irvine, Irvine, CA 92697-3900 USA; 2 Department of Pharmaceutical Sciences, School of Pharmacy and Pharmaceutical Sciences, University of California Irvine, Irvine, CA USA; 3 Center for Virus Research, University of California Irvine, Irvine, CA USA; 4 Institute for Immunology, University of California Irvine, Irvine, CA USA

**Keywords:** Cryoelectron microscopy, Innate immunity, Bacteriophages

## Abstract

Reporting in *Nature Communications*, Huo and colleagues provide three-dimensional structures of a bacterial immune defense system called Gabija. This work builds on recently published structural and functional studies and contributes strong evidence that protein assembly formation is essential for antiviral function.

The ability to recognize and respond to pathogenic invaders through immune defense is essential to the health and survival of all cellular life on Earth. To date, much of the biomedical research carried out to understand immunity has focused on humans and their interactions with microbes (with good reason). Recently, however, researchers within the immunity field have begun to take more of an interest in understanding the defense strategies of the microbes themselves.

Bacteria are prone to infection by bacteria-specific viruses called bacteriophages, or phages. Many of these phages enact a lytic cycle: phages infect the cell, utilize bacterial host machinery to replicate, and lyse the host cell, in turn expanding the population of phages and going on to infect other bacteria. Aside from the lytic cycle, phage may also enact lysogeny, a process by which phage genetic material integrates into the host genome, remaining silently in the cell throughout a host replication cycle in order to later proliferate and expand. As phages have evolved to effectively infect and lyse bacteria, bacteria have also evolved defense systems to fight back against pathogens. The most widely known and well-studied among these include restriction-modification and CRISPR-Cas9 systems, which are now utilized as prominent biotechnological tools. In the last several years, the list of known antiphage defense systems has expanded to include dozens of unique immune defense strategies that promote the survival of bacterial populations infected by phage^1–4^. One of these recently discovered defense systems is named Gabija, taking its name from the Lithuanian goddess of fire, which degrades phage DNA to restrict viral replication^1^.

Gabija is a widespread antiviral system, found in as many as ~15% of bacteria and archaea for which genome sequences are available^5^. The function of this defense pathway involves two separate proteins, GajA and GajB, both of which are essential for protection against phage^1^. Despite previous studies revealing a potential role for direct interactions between GajA and GajB as well as identification of critical amino acids required for efficient phage nucleic acid degradation, the underlying mechanistic details of Gabija defense have remained partly mysterious^6–8^. The structural basis of Gabija immunity is revealed in a new manuscript published in *Nature Communications* by Huo and colleagues where the authors used a cryo-EM (electron microscopy) approach to get a better picture of the complex interactions between GajA and GajB and how these interactions may drive nuclease activity^9^. Previous work has moved toward clarifying the structure of each component, the structure of the complex, and the activity and includes recently published structures of the GajAB complex, also determined using X-ray crystallography and cryo-EM^10^. The data presented by Huo et al. corroborate many of these findings regarding structural homology, subunit contacts, and enzymatic ATPase and nuclease activities, and they further explore substrate specificity for both DNA and nucleotides^9^.

Gabija is composed of a 4:4 ratio of the system’s two encoded proteins, GajA and GajB, that form a heterooctameric complex (GajAB) through which immune protection stems (Fig. 1)^8–10^. Each of these proteins has distinct roles that are currently understood in the GajAB complex: GajA is an endonuclease responsible for the DNA cleavage activity and GajB regulates GajA specificity and activity^6^. Comparisons to proteins homologous to GajA, namely OLD (overcoming lysogenization defect) family nucleases, point toward the protein’s function in defense against lysogenic phage cycles. OLD nucleases, like GajA and some effector domains from the retron antiphage systems, are composed of an N-terminal ATP-binding cassette (ABC)-family ATPase and a C-terminal Toprim domain which houses the catalytic center for nucleic acid cleavage^11,12^. GajB has structural homology with ATPases of the UvrD/PcrA/Rep-like SF1 helicase family although evidence for helicase-like unwinding of DNA by GajB has not been observed. Huo et al. highlight key residues that form the interfaces between GajA-GajA, GajB-GajB, and GajA-GajB subunits and using chromatographic approaches further confirm that the octamer is assembled first from tetrameric GajA subcomplexes and monomeric GajB, previously assessed by Oh et al.^8^. Interestingly, an entire domain of GajB was unobserved in the cryo-EM data and also shown not to be essential for GajAB complex formation and the role for this domain in influencing phage protection activity is unlear^9^. While GajB makes few homodimeric contacts it is instead stabilized in GajAB through an interface with the ATPase domain of GajA.

**Fig. 1:**
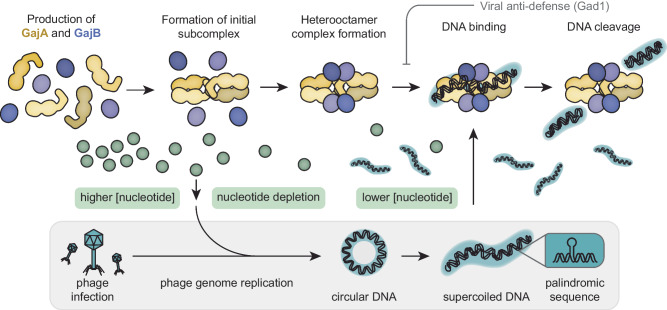
Model of Gabija antiviral defense. GajA subunits first form a tetramer to which GajB subunits bind in order to fully assemble into Gabija heterooctameric complexes. Gabija scouts for phage DNA and recognizes a palindromic sequence. Through nuclease activity, Gabija degrades phage DNA to counter infection and limit the formation of infectious phage particles. A role for nucleotide depletion in regulating the activation of Gabija is proposed and it was recently shown that phage can also counter Gabija through direct protein-protein interactions with anti-defense protein Gad1. GajA, gold; GajB, purple; phage and phage DNA, blue; free nucleotides (NTP/dNTPs), green.

The biochemical data from Huo et al. further supports that nucleotide depletion contributes to the regulation of the complex, a phenotype that had been reported elsewhere^6,7^. Mounting evidence suggests that DNA degradation is inhibited when ATP/GTP (dATP/dGTP) concentrations are high but that this inhibition is released as nucleotide concentration drops below a threshold. Mechanistically this could be a regulatory feature wherein the depletion of free nucleotides driven by phage DNA replication (incorporation of the nucleotide into nucleic acid) is a trigger for the activation of Gabija. Additionally, the authors provide more direct evidence for ATPase/GTPase activity of both GajA and B subunits as well as the full GajAB complex and show that these activities are related to DNA specificity and nuclease function. Huo et al. propose that Gabija demonstrates preferential specificity for circular, supercoiled DNA over linear DNA^9^. DNA constructs used in their experiments contained a palindromic motif, which may reveal the putative role of nucleic acid hairpins in the mechanism of recognition by the Gabija complex. Structural analysis by the authors identified a patch of positively charged residues in GajA (lysine patch 1) which contributed to this specificity for circular, supercoiled DNA^9^. Specificity for the supercoiled DNA, however, was dependent not only on this residue patch but also on interactions with GajB, such that when GajA interacted with GajB, supercoiled DNA was increasingly favored as a cleavage substrate.

The structural and functional characterization reported by Huo et al. joins other recent works that open the doors to a new understanding of the Gabija defense system. In light of the evolutionary arms race between bacteria and these invading phages, this discovery of the active Gabija complex conformation highlights that phages may likewise have evolved to evade host defense systems. One such evasion tactic has already been detailed by Antine et al. with the phage protein Gad1 (Gabija anti-defense 1) enveloping the Gabija complex to inactivate it by preventing nucleic acid binding interactions^10,13^.

The structure of the GajAB octamer may allow for the development of therapeutics that take advantage of the specific DNA cleavage activity of Gabija and could lead to the development of biotechnological tools, as is the case for the RM and CRISPR-Cas9 systems. Yet more remains to be understood regarding the role of each subunit within the overall context of defense. GajB was highlighted by the authors to cleave ATP, but it remains to be confirmed whether this ATP consumption may lead to nucleotide depletion to the degree that even host replication may be affected. This would lead to abortive infection, in which the host may have reduced growth or may essentially sacrifice itself to protect neighboring cells and further halt phage replication. If it is the case that a pathogenic bacterium that utilizes the Gabija system is found in patients, Gabija-driven abortive infection through cell death may be a plausible point of a therapeutic intervention. Also, while a variety of nucleotides were shown to be suitable substrates for Gabija, the relevance of this partly non-selective activity and related influence on nucleic acid recognition and degradation must be further explored. The structural basis of Gabija-DNA interactions remains to be shown but given that the platform for investigating the mechanisms linking ATPase and nuclease activities at the atomic level is now established by Antine and colleagues and Huo et al. (as well as preprinted by Fu et al.) it is only a matter of time before these details are brought to light^14^.
